# Supplementary material for: The LIM Protein Ajuba Augments Tumor Metastasis in Colon Cancer
Source: Cancers (Basel). 2020 Jul 15;12(7):1913. doi: 10.3390/cancers12071913 (PMC7409172; doi:10.3390/cancers12071913)
Supplement: Supplementary file 1 [file cancers-12-01913-s001.pdf]

Supplementary Materials: The LIM Protein Ajuba Augments Tumor Metastasis in Colon Cancer

Noëlle Dommann, Daniel Sánchez-Taltavull, Linda Eggs, Fabienne Birrer, Tess Brodie, Lilian Salm, Felix Alexander Baier, Michaela Medová, Magali Humbert, Mario P. Tschan, Guido Beldi, Daniel Candinas and Deborah Stroka

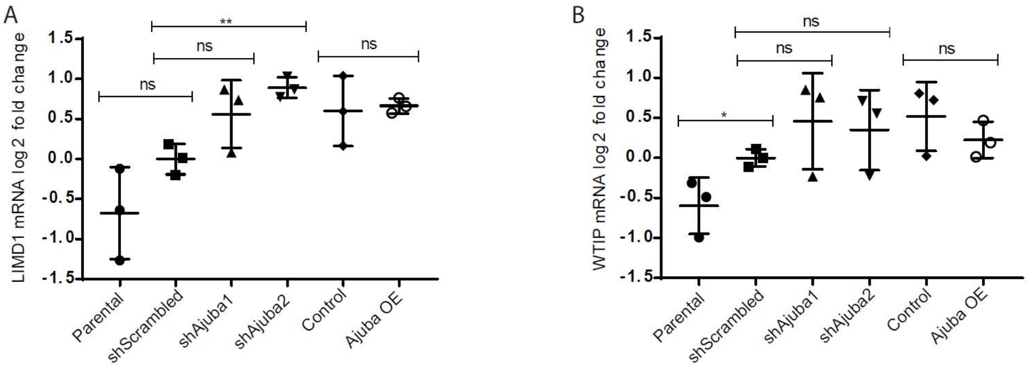

Figure S1. (**A**) LIMD1 mRNA expression levels measured by RT-qPCR in SW480 cells after Ajuba KD and OE. (**B**) WTIP mRNA expression levels measured by RT-qPCR in SW480 cells after Ajuba KD and OE.

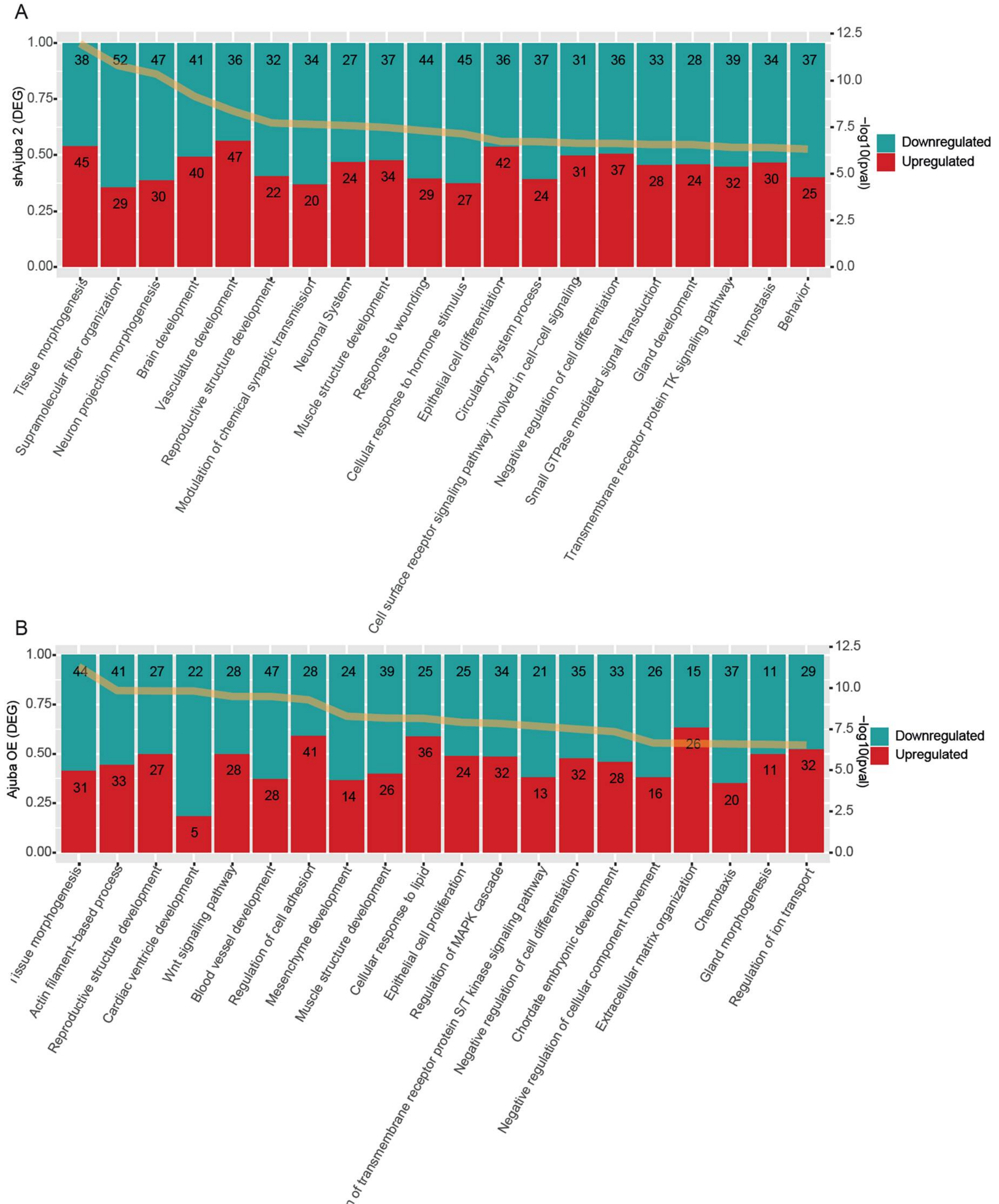

Figure S2. (**A**) Graph showing pathway enrichment analysis of significantly differentially expressed genes between shAjuba2 and its shScrambled. Pathway enrichment has been done with Metascape. The 20 family of pathways with the lowest -log10 adjusted p value are depicted as well as gene ontology term they are involved. The yellow line across the figure represents the calculated p value. (**B**) Graph showing pathway enrichment analysis of significantly differentially expressed genes between Ajuba OE and its control. Pathway enrichment has been done with Metascape. The 20 family of pathways with the lowest -log10 adjusted p value are depicted as well as gene ontology term they are involved. The yellow line across the figure represents the calculated p value.

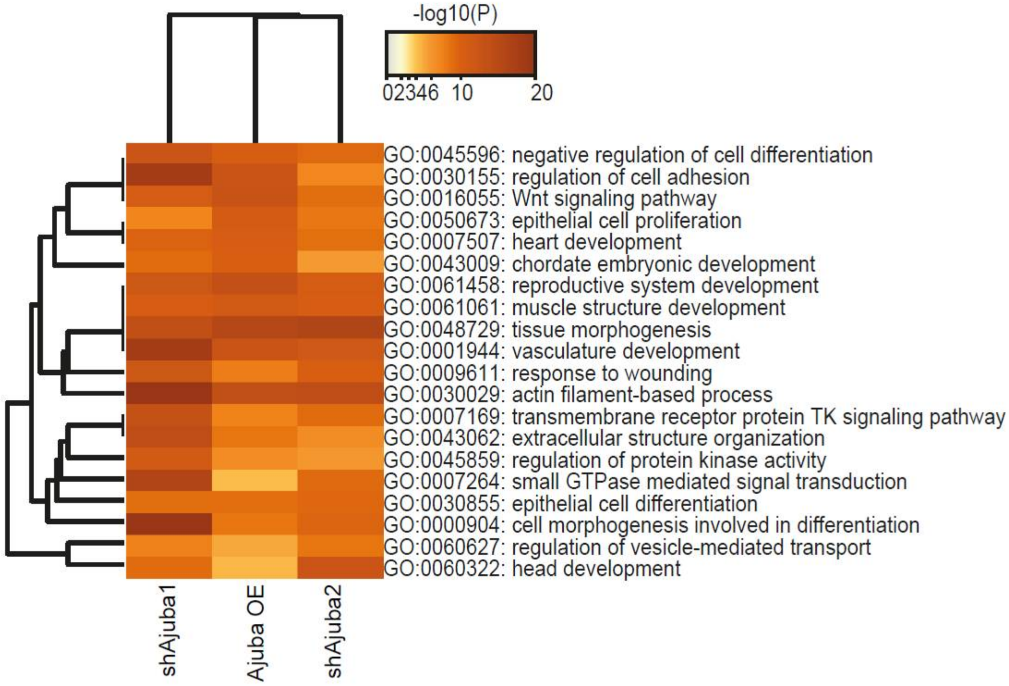

Figure S3. Heat map showing commonly altered pathways from shAjuba1, shAjuba2 and Ajuba OE showing their P value and in which Gene ontology term they are involved. Done with Metascape using multiple gene list function. The 20 family of pathways with the lowest -log10 adjusted p value are depicted.

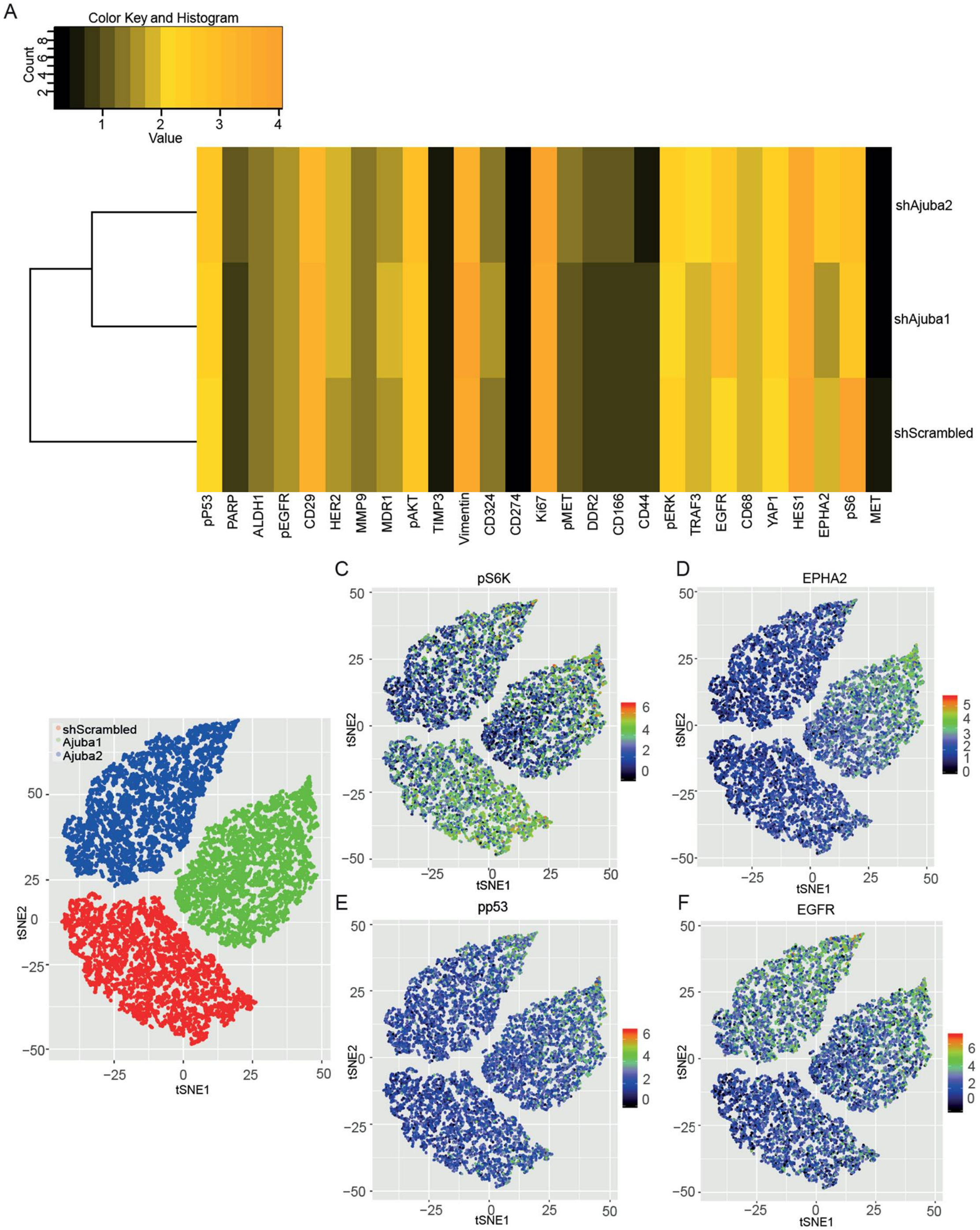

Figure S4. (**A**) Heat map representing the median expression of the SW480 colon cancer cell line where Ajuba has been previously KD. (**B**) t-SNE plot of SW480 cancer cell line with Ajuba KD and shScrambled control clustering has been done according to heavy metal barcoding. (**C**–**G**) *t*-SNE plot showing expression level of different antibodies.

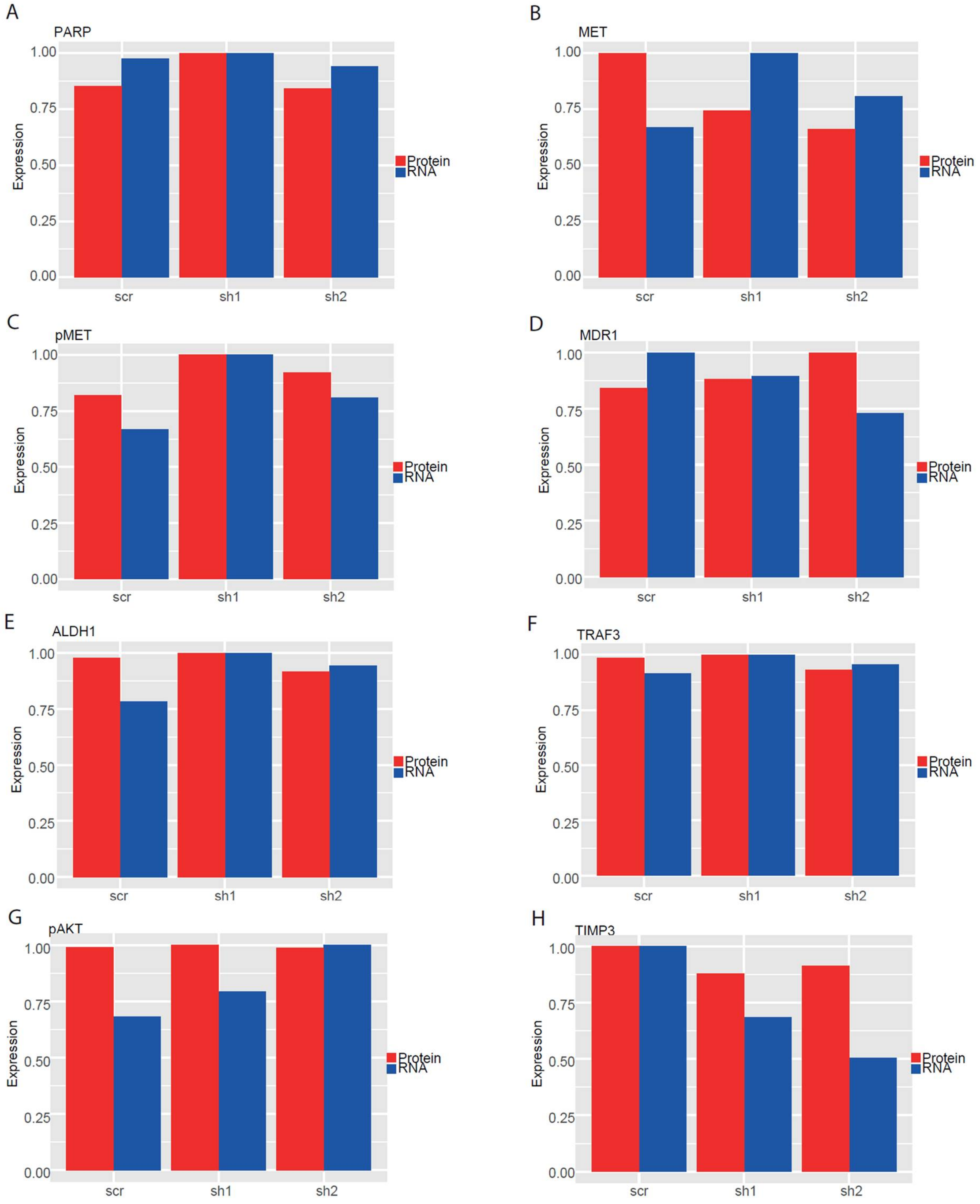

Figure S5. (**A**–**H**) Bar graphs displaying correlation between protein and RNA expression. Averaged and normalized RPM values of the RNA-seq and the arcsinh transformed CyTOF data were computed independently and the rescaled values used to compare expression.

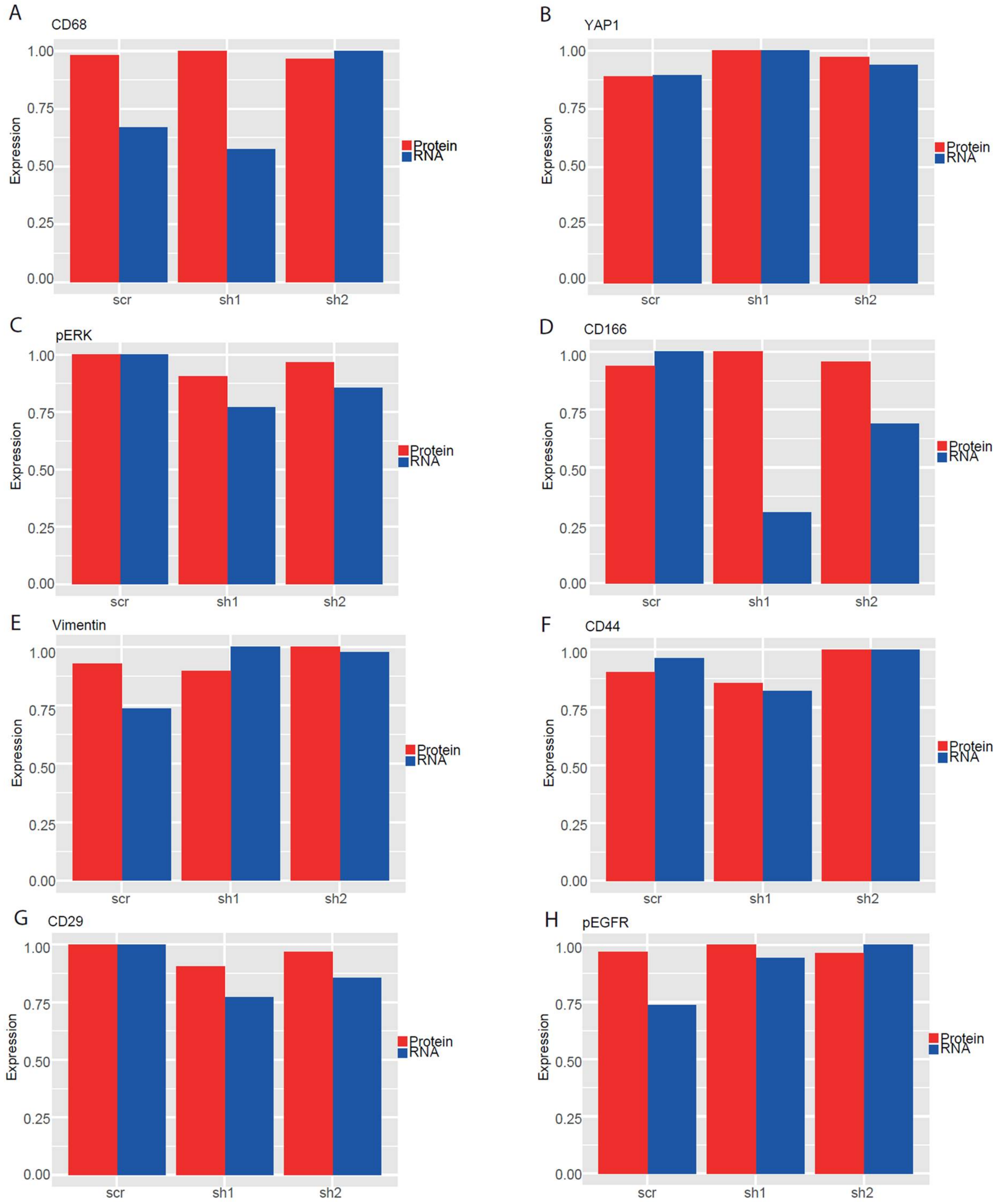

Figure S6. (**A**–**H**) Bar graphs displaying correlation between protein and RNA expression. Averaged and normalized RPM values of the RNA-seq and the arcsinh transformed CyTOF data were computed independently and the rescaled values used to compare expression.

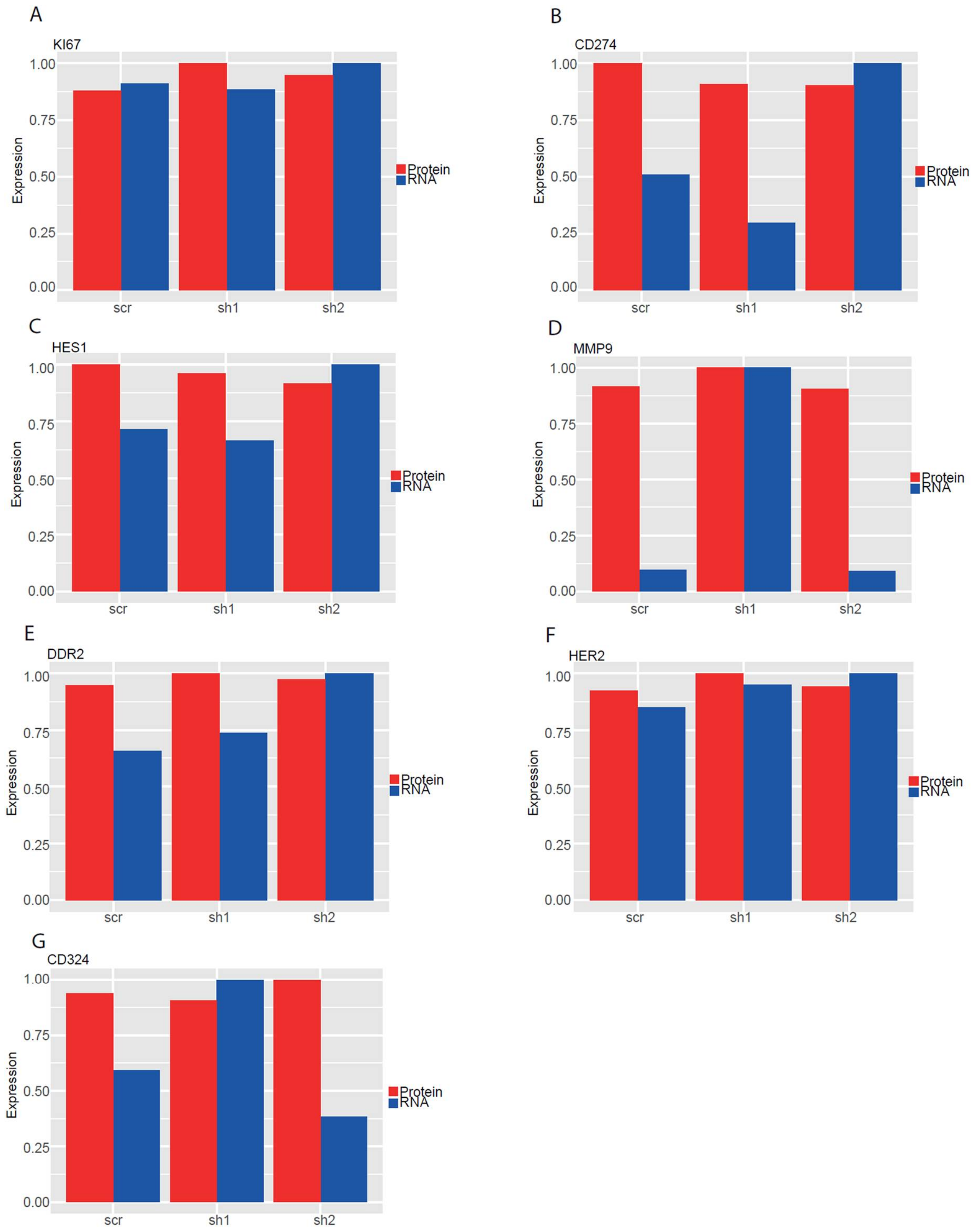

Figure S7. (**A**–**H**) Bar graphs displaying correlation between protein and RNA expression. Averaged and normalized RPM values of the RNA-seq and the arcsinh transformed CyTOF data were computed independently and the rescaled values used to compare expression.

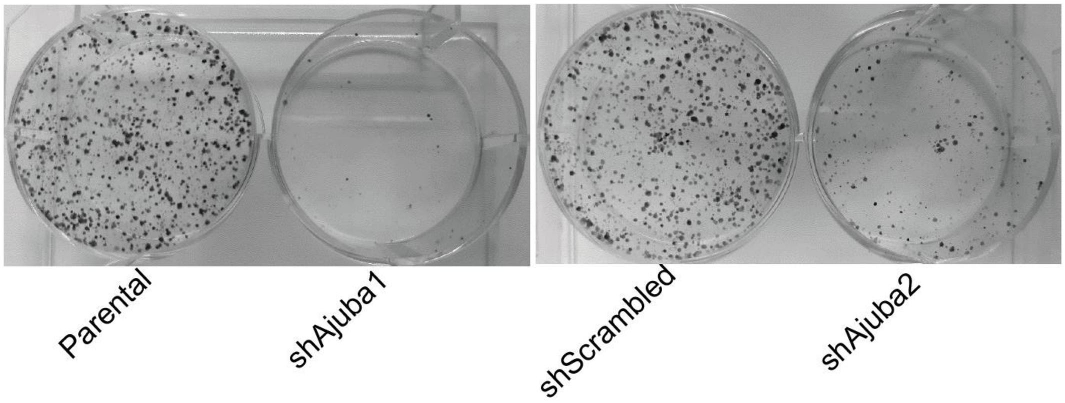

Figure S8. Pictures of colony formation assay in 6-well plates, after being stained with crystal violet. One representative well per condition is displayed.

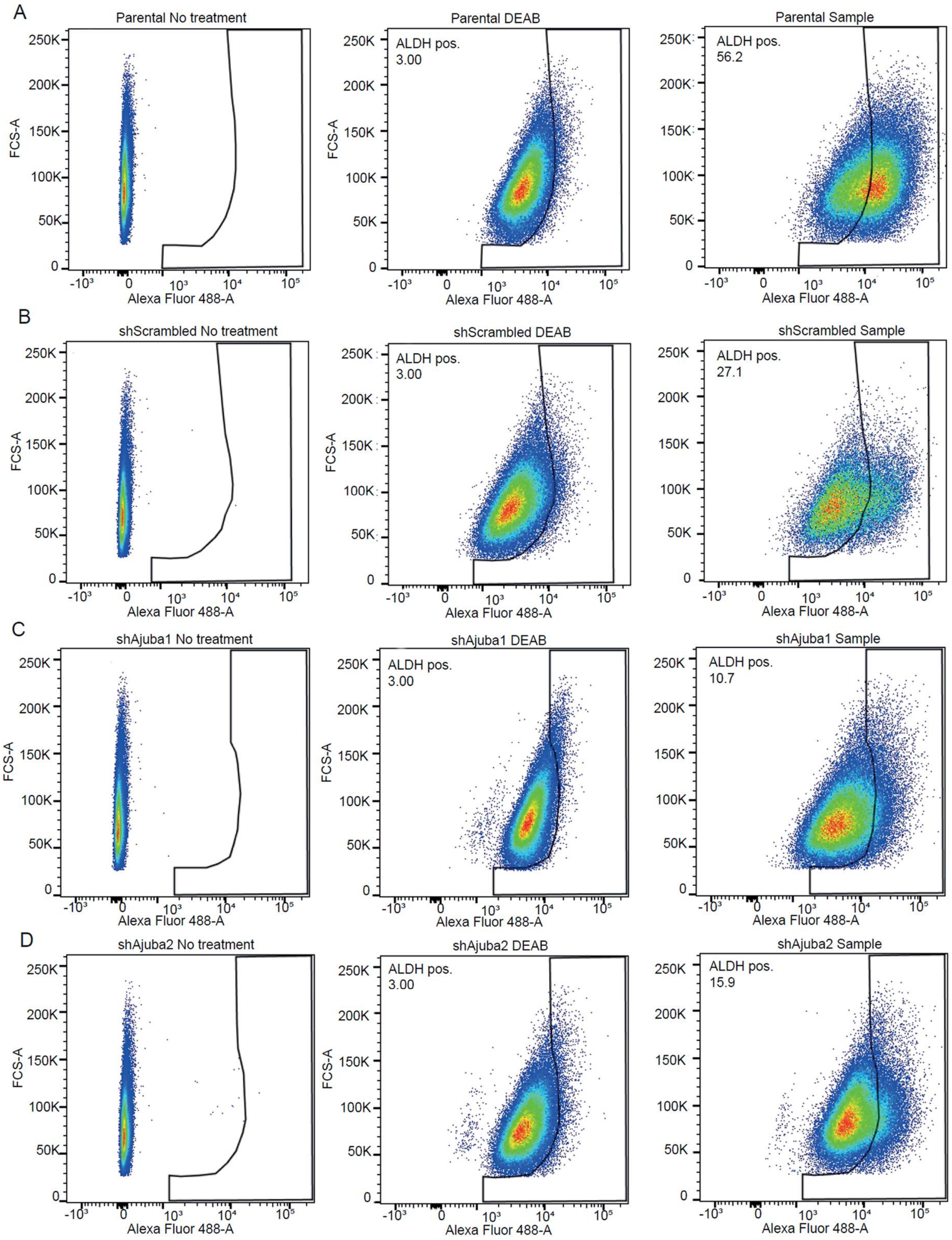

Figure S9. (**A**–**D**) FACS plots showing gating strategy to identify ALDH positive cells. The cells were gated as negative according to the DEAB treated samples which accounts for 3% positive cells in all negative controls. The same gate was applied to untreated samples and to the test sample in order to determine how many cells are ALDH positive (*N* = 3, representative sample is displayed to show gating strategy).

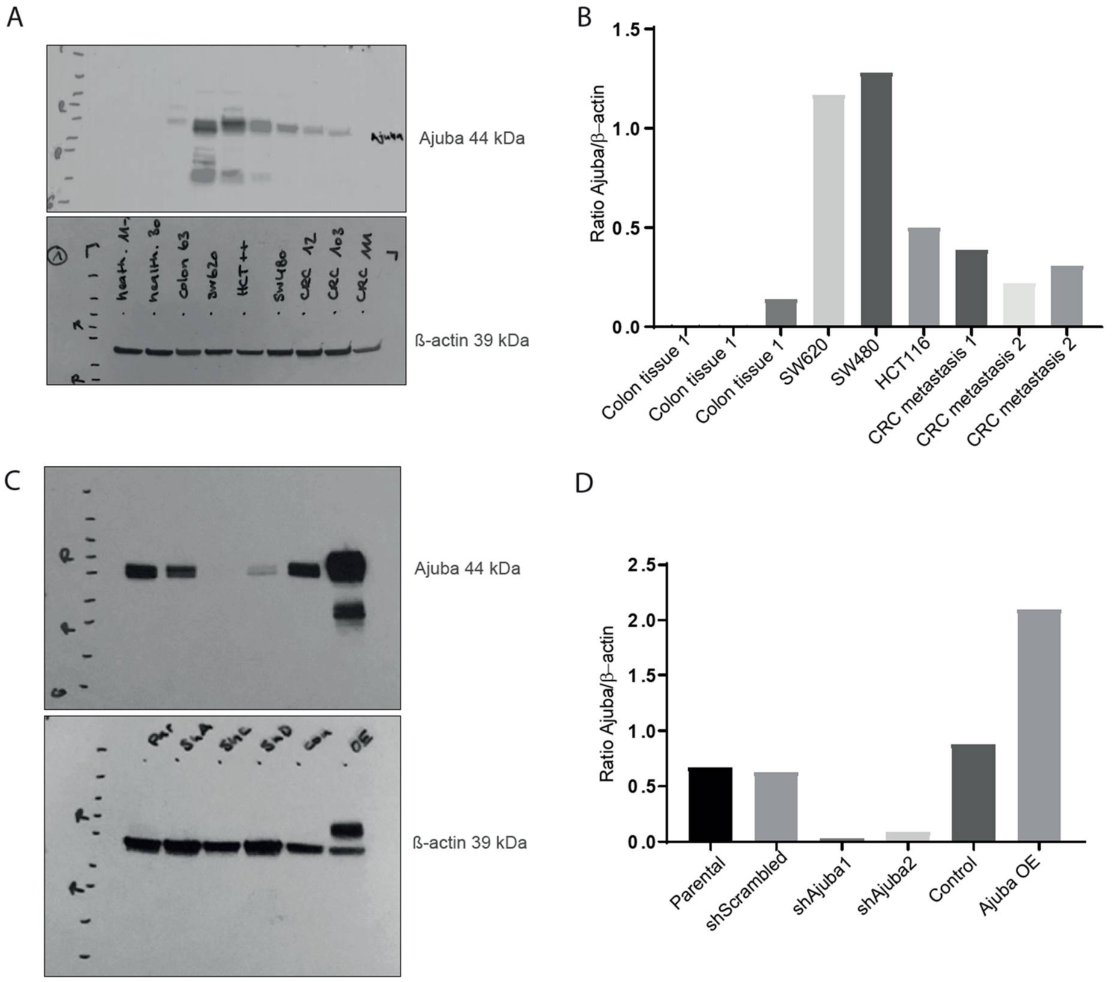

Figure S10. (**A**) Whole Western blot membrane of human control colon cancer cell lines and colon metastasis in the liver (as depicted in Figure 1B). (**B**) Western blot quantification using ImageJ and calculating the Ajuba/β-actin ratio. (**C**) Whole Western blot membrane of SW480 colon cancer cell line in which Ajuba has been KD and OE. (As depicted in Figure 2B). (**D**) Western blot quantification using ImageJ and calculating the Ajuba/β-actin ratio.

Generating CRISPR/Cas9 mediated Ajuba KO in cancer cell line

We used the protocol established by Ren et al.[1]. In brief, we designed two different gRNAs that target either exon 1 or exon 2 of the murine Ajuba sequence. gRNA design was made with the online tool of the Zangh lab (http://tools.genome-engineering.org/). The gRNAs were cloned into the vector pSpCas9 (BB)-2A-GFP (px458, purchased from addgene.org).

gRNA 1: 5′–CACCGAGCCGAATCCCACGGACCGA –3′ … (followed by PAM sequence) (targets exon 1 of Ajuba).

gRNA 2: 5′– CACCGACCTTTGTTGCACTTGATAC–3′ … (followed by PAM sequence) (targets exon 2 of Ajuba)

(Restriction sites in red, additional guanine in light blue)

Plasmids containing either gRNA1 or gRNA2 were then transfected into RIL-175 cells using Lipofectamine® 3000 (Thermo Fisher Scientific). Successful transfection was confirmed after 2 days by fluorescence microscopy (Leica DMI4000B) for GFP (A). One part of the transfection positive cells was then sorted by fluorescence-activated cell scanning for GFP positivity (BD FACSAria™ III) in bulk into tubes and used for total protein isolation. The absence of Ajuba protein in gRNA1 and gRNA2 transfected cells was then confirmed by western blot (B). The other part of the transfected cells were sorted as single cells into a 96-well plate for clonal selection. However, the single cell sorted cells with Ajuba CRISPR knock-out did not grow out colonies, and also bulk sorted transfected cells died and detached from the plates after 4 days of culture.

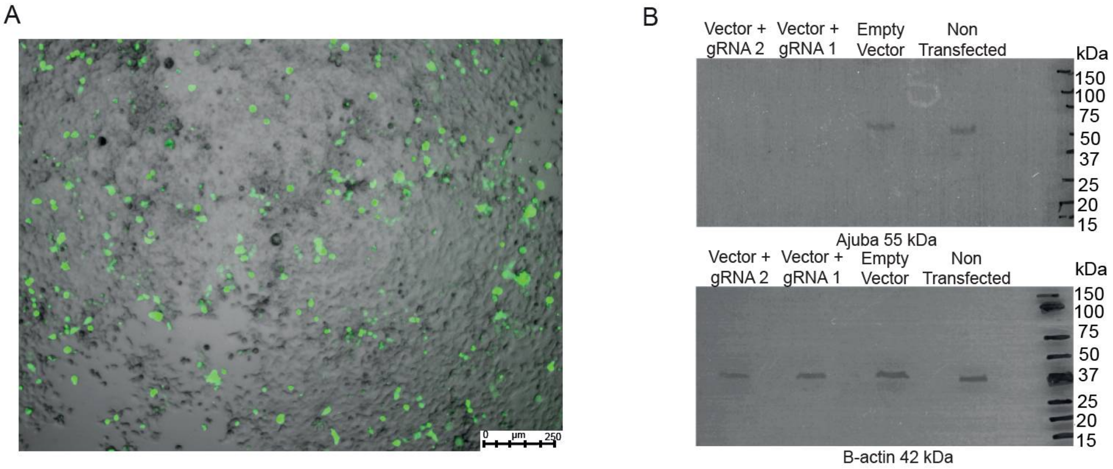

Figure S11. (**A**) CRISPR/CAS9) transfected RIL-175 cells. GFP as positive control for efficient transfection. (**B**) Western blots to proof KO of Ajuba protein.

Reference

1. Ran, F.A.; Hsu, P.D.; Wright, J.; Agarwala, V.; Scott, D.A.; Zhang, F. Genome engineering using the CRISPR-Cas9 system. *Nat. Protoc.* **2013**, *8*, 2281–2308.

© 2020 by the authors. Licensee MDPI, Basel, Switzerland. This article is an open access article distributed under the terms and conditions of the Creative Commons Attribution (CC BY) license (http://creativecommons.org/licenses/by/4.0/).
